# Viral metagenomic analysis of fecal samples from *Bos grunniens* on the Qinghai-Tibet Plateau reveals novel picornaviruses and diverse CRESS-DNA viruses

**DOI:** 10.3389/fcimb.2025.1719300

**Published:** 2026-01-07

**Authors:** Jiaheng Chen, Ga Gong, Xiaodong Su, Xiaofei Song, Jingyue Zhang, Ping Wu, Hua Wang, Tongling Shan, Wen Zhang

**Affiliations:** 1 Department of Laboratory Medicine, School of Medicine, Jiangsu University, Zhenjiang, Jiangsu, China; 2 Animal Science College, Tibet Agriculture and Animal Husbandry University, Nyingchi, Tibet, China; 3 Tianjun County Center for Disease Control and Prevention, Tianjun, Qinghai, China; 4 Department of Biology, Shenzhen MSU-BIT University, Shenzhen, Guangdong, China; 5 Shanghai Veterinary Research Institute, Chinese Academy of Agricultural Sciences, Shanghai, China

**Keywords:** CRESS-DNA viruses, fecal virome, metagenomics, Picornaviridae, Qinghai-Tibet Plateau, yak

## Abstract

**Introduction:**

The Qinghai–Tibet Plateau (QTP), one of the most extreme environments on Earth, provides a unique natural setting for exploring viral diversity and evolution under conditions of high altitude, hypoxia, and intense ultraviolet radiation. The yak (*Bos grunniens*), a key endemic ruminant species of the QTP, plays an essential ecological and economic role, yet its fecal virome remains poorly characterized.

**Methods:**

In this study, we analyzed 43 yak fecal samples collected from Yushu, Qinghai Province, and constructed nine metagenomic libraries to investigate the composition, diversity, and phylogenetic characteristics of the yak fecal virome.

**Results:**

Metagenomic sequencing generated approximately 463 million raw reads, of which 2.87 million were classified as viral. The viral reads in the sequenced libraries were primarily composed of single-stranded DNA viruses (92.46%), particularly members of *Smacoviridae, Circoviridae*, and *Genomoviridae*, whereas RNA viruses such as *Picornaviridae* accounted for a minor fraction (0.71%). Phylogenetic analyses revealed that several circular single-stranded DNA (CRESS-DNA) virus and picornavirus genomes share high similarity with known ruminant-associated viruses, while forming independent evolutionary clades, suggesting potential cross-species transmission among plateau animals. The large-scale divergence within *Smacoviridae* further reflects extensive lineage expansion under the plateau’s extreme environmental pressures.

**Discussion:**

Compared with our previous yak virome study, this work provides independent and complementary insights into the genomic and evolutionary characteristics of key viral taxa. Overall, our findings expand the genomic landscape of the yak fecal virome and highlight the Qinghai–Tibet Plateau as an important reservoir for exploring viral diversity, evolution, and host–environment interactions in extreme ecosystems.

## 1 Introduction

The Qinghai-Tibet Plateau, known as “the Roof of the World“, has an average elevation exceeding 4, 000 meters. Its extreme environmental conditions—characterized by high altitude, hypoxia, and intense ultraviolet radiation—have shaped its unique biotic communities and ecosystems (Päckert et al., 2020). However, this distinctive plateau ecosystem is confronted with significant challenges, which are reflected not only in the thawing of glaciers and permafrost but also in the dynamic balance of biodiversity (Qian et al., 2023). Among the endemic species of the Qinghai-Tibet Plateau, the yak (*Bos grunniens*), a unique bovine species, has evolved distinct physiological traits following long-term adaptation to the plateau environment. These traits include increased red blood cell concentration in the blood and enhanced mitochondrial energy metabolism, among others (Xue et al., 2021). Meanwhile, molecular genetic studies have demonstrated that, in high-altitude regions, the adaptive evolution of genes related to energy metabolism provides the yak with favorable survival conditions (Zhang Z. et al., 2016). In addition, recent studies have indicated that the intestinal virome within the host regulates microbial community structure, immune responses, and nutrient metabolism (Zhang X. et al., 2016; Jia et al., 2021; Ji et al., 2021; Sha et al., 2021). Nevertheless, in recent years, most microbial research on plateau animals has focused on bacterial communities, while studies investigating the genetic diversity, functions, and evolutionary mechanisms of intestinal viruses remain extremely limited.

In recent years, cross-species transmission events of zoonotic viruses have occurred frequently, making the surveillance of wildlife viromes imperative (Zeller et al., 2016; Acevedo et al., 2021). The yak not only serves as a primary economic resource for plateau herdsmen but also has the potential to act as a hub for viral transmission (Otieno et al., 2021a). Furthermore, extreme plateau climates—such as intense ultraviolet radiation and seasonal freeze-thaw cycles—may select for viruses tolerant to extreme environments. Following transmission, these viruses could pose threats to low-altitude ecosystems and human health (Su et al., 2016; Luo et al., 2023).

Traditional virological research primarily relies on the *in vitro* culture of host cells. However, the efficiency of viral isolation and culture remains low under the extreme harsh weather conditions at high altitudes. Under such circumstances, the capsid protein structure of yak enteric viruses may exhibit enhanced stability, which further increases the difficulty of traditional detection techniques. Viral metagenomics technology—integrating culture-independent methods, high-throughput sequencing, and bioinformatics analysis—enables more effective characterization of viral composition and viral-host interactions. Notably, this technology has successfully identified a large number of novel viruses in several extreme regions (Paez-Espino et al., 2016; Liu et al., 2021; Satam et al., 2023; Kim et al., 2024), thereby revealing the adaptive capabilities of viruses in extreme environments.

In this study, we will employ metagenomic approaches to continuously explore the diversity of the animal fecal virome, with a focus on viruses belonging to the *Picornaviridae* family and single-stranded circular DNA viruses (CRESS-DNA viruses). The *Picornaviridae* family comprises non-enveloped viruses with icosahedral capsids. These viruses possess a single-stranded positive-sense RNA genome, with a length ranging from approximately 6.7 to 10.1 kbp. Notably, the family includes over 80 genera, encompassing numerous human and animal-associated pathogens such as *Enterovirus* (Li M-L. et al., 2021), *Aphthovirus* (Li K. et al., 2021), *Hepatovirus* (Zell et al., 2017; Shin and Jeong, 2018). Additionally, picornaviruses have a broad host range and are transmitted primarily via the fecal-oral route to infect mammals, birds, and other organisms, thereby causing a series of gastrointestinal diseases (Zhang Z. et al., 2016). Single-stranded circular DNA viruses (CRESS-DNA viruses) are defined by their ssDNA genome and encode conserved replication-associated proteins (Rep) and capsid proteins (Cap) (Zhao et al., 2019). Several families of ssDNA viruses have been confirmed to infect eukaryotes, including *Circoviridae*, *Smacoviridae*, and *Genomoviridae*; these viruses primarily infect mammals, vertebrates, fungi, plants, and other related organisms. Characterized by strong ecological penetration ability, this group of viruses can survive for extended periods under extreme conditions (Kinsella et al., 2020; Fehér et al., 2021; Capozza et al., 2022). Thus, analyzing the composition of mammalian intestinal viromes is critical for elucidating their relationships with human health and disease.

However, current research on the viromes of high-altitude animals remains limited, and studies specifically analyzing the fecal virome of yaks on the Qinghai-Tibet Plateau are even scarcer. To investigate the diversity of fecal-associated viruses in high-altitude animals and address the gap in viral research under the extreme plateau environment, we collected 43 fecal samples from yaks on the Qinghai-Tibet Plateau and constructed 9 metagenomic libraries to perform bioinformatics analysis of potential viruses.

## 2 Material and methods

### 2.1 Collection and preparation of yak samples

To investigate the fecal viruses of yaks in high-altitude regions, 43 fresh yak fecal samples were collected in Yushu, Qinghai Province, in October 2024 (**Supplementary Figure 1**). Immediately after the yaks defecated, fecal samples were collected using sterile swabs, placed into sterile containers, and transported to the laboratory on dry ice. All yaks were captive-bred by local herdsmen and were clinically healthy with no signs of disease. For each sample, 100 mg of feces was resuspended in Dulbecco’s phosphate-buffered saline (DPBS). The mixture was centrifuged at 15, 000×g and 4 °C for 10 minutes; the supernatant was then collected into 1.5 mL centrifuge tubes and stored at -80 °C for subsequent nucleic acid extraction (Xi et al., 2023). All procedures in this study were conducted in accordance with the guidelines of the biosafety level 2 (BSL-2) laboratory and were approved by the Medical Ethics Committee of Jiangsu University.

### 2.2 Nucleic acid extraction and library preparation

To optimize sequencing efficiency while maintaining a broad survey of viral diversity, the supernatants of the 43 fecal samples were separately aspirated and pooled into 9 sample pools, with each pool having a volume of approximately 500 μL. Subsequently, eukaryotic cells were removed by passing the pools through a 0.45 μm syringe filter (Millipore). After centrifugation at 13, 000×g and 4 °C for 5 minutes, 166.5 μL of the filtrate was collected. Next, the filtrate was treated with a mixture of DNase (deoxyribonuclease), RNase (ribonuclease) and Benzonase to digest unprotected nucleic acids at 37 °C for 60 minutes. Nucleic acids were extracted using the QIAamp Viral RNA Mini Kit (QIAGEN) according to the manufacturer’s instructions. Viral nucleic acids were then reverse-transcribed into cDNA using a reverse transcription kit (SuperScript IV, Invitrogen) with 6 random primers; the resulting product was denatured at 95 °C for 2 minutes and then placed on ice for at least 2 minutes (Qian et al., 2024). Subsequently, Klenow fragment polymerase (NewEngland BioLabs) was used to perform a single cycle of DNA synthesis, converting all single-stranded DNA (ssDNA) into double-stranded DNA (dsDNA) for library construction. Finally, libraries were constructed using the Nextera XT DNA Sample Preparation Kit (Illumina) and sequenced on the Illumina NovaSeq 6000 platform with 150 bp paired-end reads (Lu et al., 2024).

### 2.3 Bioinformatics analysis

Software provided by Illumina was used to pair the 150 bp paired-end reads generated by sequencing. Data processing was performed using an in-house analysis pipeline on a 32-node Linux cluster. First, the genome sequence of *Bos grunniens* (GCA_005887515.3) was downloaded from the NCBI database. Bowtie2 v2.5.0 was used for alignment to remove potential host sequences from the 9 libraries (Langmead and Salzberg, 2012). Trim Galore v0.6.7 was employed to remove primer sequences and low-quality reads (parameters: –phred33 –length 100 –stringency 3 –paired), thereby retaining core high-quality sequence fragments (Krueger et al., 2023). Subsequently, MEGAHIT v1.2.9 (parameter: –min-contig-len 200) was used for *de novo* assembly of these reads to generate contigs (maximum contiguous sequences) and singlets (single-copy sequences) (Li et al., 2016). BLASTx analysis was performed on the assembled contigs and raw reads separately using DIAMOND v2.1.12 with default parameters (Buchfink et al., 2021); each sequence was aligned against the viral protein database in NCBI, and an E-value threshold was set to filter potential viral sequences, with an E-value cutoff of < 10^-5^. To further eliminate potential false-positive viral sequences, the candidate viral hits were re-aligned against an in-house non-viral non-redundant (NVNR) protein database. This database was compiled using non-viral protein sequences extracted from the NCBI non-redundant (nr) database, based on taxonomic annotations excluding the Virus Kingdom. Finally, the DAA-format results output by DIAMOND v2.1.12 were imported into MEGAN v7.1.1 and converted to RMA format for viral community analysis and subsequent phylogenetic analysis (Huson et al., 2016).

### 2.4 Viral community analysis

Statistical analyses were performed using R v4.4.2 and MEGAN v7.1.1. With MEGAN, the compositional analysis results of the 9 sample pools were standardized and compared (Huson et al., 2007). Viral community structure and richness were visualized using the ggplot2 (Wickham, 2016) and pheatmap (Kolde, 2010) packages in R v4.4.2.

### 2.5 Metagenomic assembly and phylogenetic analysis

Contigs with significant similarity to BLASTx hits were selected, and those with a sequence length > 500 bp were analyzed. Each contig was used as a reference to map raw data from its original barcode, with the *Low sensitivity/Fastest* parameter set in Geneious Prime v2024.0.5, thereby obtaining complete or nearly complete contigs similar to known viruses. In Geneious Prime v2024.0.5, specific open reading frames (ORFs) with a minimum length of 400 bp and ATG as the start codon were predicted. These ORFs were aligned and validated against viral proteins in the GenBank database. ORF annotation was performed by matching against CDD-62456 PSSMs from the Conserved Domain Database (https://www.ncbi.nlm.nih.gov/Structure/cdd/wrpsb.cgi) (Marchler-Bauer et al., 2017). Phylogenetic analysis was conducted using three types of sequences: the predicted protein sequences of viruses identified in this study, the sequences with the highest identity retrieved from NCBI’s GenBank database via BLASTx matching (E-value cut-off < 10^-5^), and the representative viral protein sequences of relevant viral subfamilies or genera. Protein sequences were aligned using MUSCLE in MEGA v11 with default parameters (Tamura et al., 2021); the aligned results were trimmed using TrimAl v1.5.0 (-automated1) to reduce noise (Capella-Gutiérrez et al., 2009). Phylogenetic trees were then constructed using IQ-TREE v2.3.6 (-m MFP –bb 1000) (Minh et al., 2020). ChiPlot (https://www.chiplot.online/) was used to visualize the conserved domains of CDD (Ji et al., 2022) and optimize the appearance of phylogenetic trees (Xie et al., 2023). All silhouettes included in the phylogenetic trees were obtained from PhyloPic (https://www.phylopic.org/).

### 2.6 Pairwise sequence identity analysis

Sequence Demarcation Tool (SDT v1.3) was used to perform pairwise identity comparisons of all target viruses at the amino acid level. For each group of sequences, the default MUSCLE option in SDT was selected for alignment (Muhire et al., 2014).

### 2.7 Quality control

Standard precautionary measures were implemented throughout all experimental procedures. Nucleic acid samples were dissolved in DEPC-treated water, and RNase inhibitor was added to the samples. During the experiment, pipette tips equipped with aerosol filters were used, and all experimental materials that came into contact with nucleic acid samples (e.g., 1.5 mL centrifuge tubes, pipette tips) were free of RNase and DNase. These measures were taken to prevent sample cross-contamination and nucleic acid degradation.

## 3 Results

### 3.1 Overview of the yak fecal virome

Through the analysis of yak feces from the Qinghai-Tibet Plateau, metagenomic sequencing was performed on 9 libraries, generating a total of 463, 733, 934 raw reads with an average GC content of 42.16% (**Supplementary Table 1**; detailed sequencing and assembly metrics). Among these reads, 2, 869, 440 were matched to viral proteins via BLASTx searches based on protein sequence identity.

**Figure 1:**
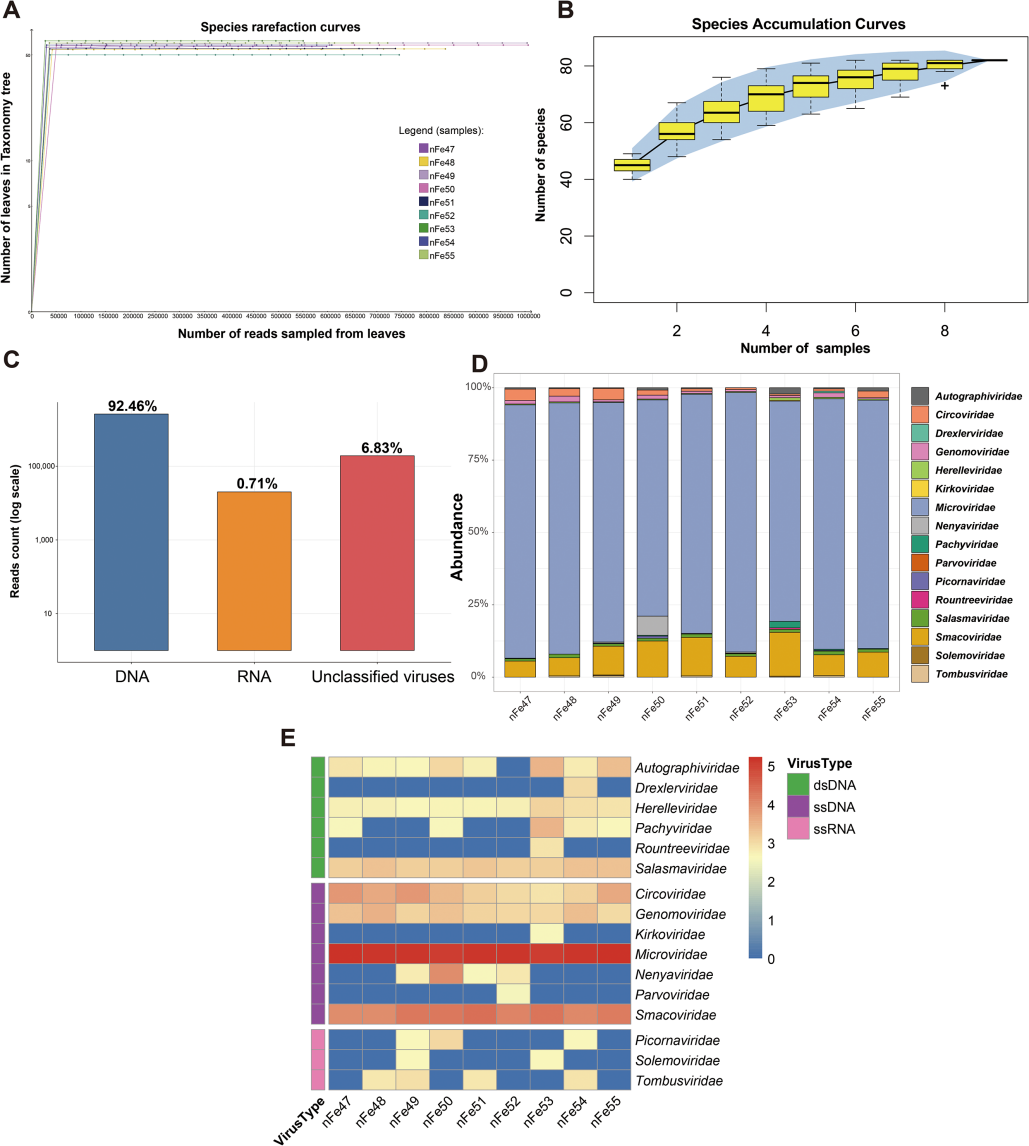
Viral diversity across the nine libraries. **(A)** Species rarefaction curves after log-scale transformation, annotated by MEGAN v7.1.1. The legend is located on the right side of the panel. **(B)** Species accumulation curve of yak fecal samples. Error bars represent the range, the blue area in the background indicates the 95% confidence interval, and plus signs (+) represent outliers. **(C)** Proportions of DNA viruses, RNA viruses, and unclassified viruses in the libraries. The y-axis is displayed on a log scale. **(D)** At the family level, a stacked bar chart of abundance was plotted, showing the proportion of different viral families in each library. The library names are at the bottom, and the legend for viral families is on the right. **(E)** Taxonomic analysis of viral reads at the family level. The heatmap of the nine libraries shows the read counts of each viral family in each library. Data are presented on a log scale with base log_10_. The legend on the right side of the heatmap indicates the names and classifications of each viral family.

Species rarefaction curves and species accumulation curves were plotted to characterize the richness of all samples. In these 9 libraries, the number of observed viral species eventually leveled off; thus, it can be inferred that the sequencing depth in this study had covered all viruses in the samples, and increasing sequencing data would not further increase viral diversity (**Figure 1A**). Additionally, the accumulation curve of viral species was found to have reached saturation, indicating that adequate and sufficient sampling of the viral community in yak feces was achieved (**Figure 1B**). Meanwhile, the accumulation curve also suggested that there might be more than 80 different viral species distributed across the 9 libraries.

Furthermore, our analysis revealed that DNA viruses accounted for a large proportion (92.46%) of the identified viruses, whereas RNA viruses only accounted for 0.71% (**Figure 1C**). The stacked abundance plot shows that *Microviridae* accounts for the largest proportion in each library, followed by *Smacoviridae* (**Figure 1D**). A heatmap showed that a total of 16 viral families were identified via metagenomic sequencing, including 6 double-stranded DNA (dsDNA) viral families, 7 single-stranded DNA (ssDNA) viral families, and 3 single-stranded RNA (ssRNA) viral families (**Figure 1E**).

To further quantitatively evaluate the viral complexity, ecological diversity metrics were calculated based on the species-level abundance profile. The Alpha diversity analysis showed that the Shannon indices of the nine libraries ranged from 2.67 to 3.08, and the Simpson indices ranged from 0.87 to 0.92 (**Supplementary Table 2**), indicating a relatively high richness and even distribution of viral species within the samples. Furthermore, Beta diversity analysis based on Bray-Curtis distance (visualized via PCoA) revealed compositional heterogeneity among the sample pools, with the first two principal coordinates explaining 45.26% and 23.39% of the variance, respectively (**Supplementary Figure 2**).

### 3.2 Picornaviridae

**Figure 2:**
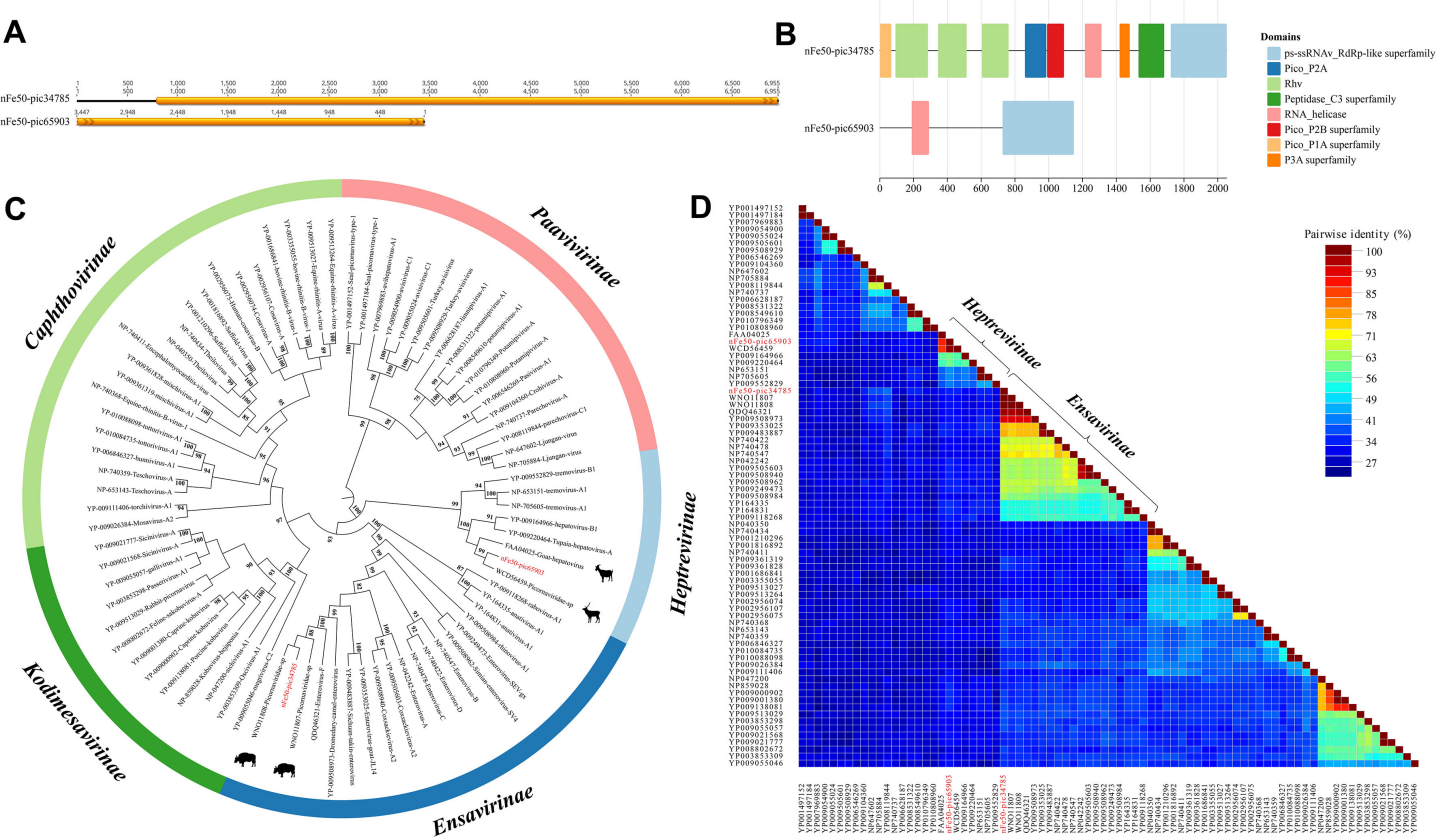
Phylogenetic Analysis of the *Picornaviridae* Family. **(A)** Two picornavirus sequences identified in yak feces. **(B)** Domain annotation plotted using ChiPlot after CDD protein function prediction; the legend on the right represents different conserved domains. **(C)** Phylogenetic tree constructed based on the RdRp protein. Color blocks represent different subfamilies, and sequences identified in this study are marked in red. Bootstrap values > 70 are displayed on the branches. **(D)** Triangular matrix heatmap showing the pairwise amino acid identity between sequences.

To further explore the composition of RNA viruses in the yak fecal virome, one nearly complete genome with an open reading frame (ORF) and one fragment encoding the RNA-dependent RNA polymerase (RdRp) protein were assembled from yak feces (**Figure 2A**). Protein functional prediction using the Conserved Domain Database (CDD) yielded annotations of the protein domains for these two viral sequences (**Figure 2B**). We constructed a phylogenetic tree based on the RdRp protein (**Figure 2C**), and the results showed that these two strains belonged to the subfamilies *Ensavirinae* and *Heptrevirinae*, respectively. Strain nFe50-pic34785 clustered with a yak-derived strain (GenBank Accession No. WNO11808), with an amino acid identity of 99.70%. Strain nFe50-pic65903 clustered with a red deer-derived strain (GenBank Accession No. WCD56459), with an amino acid identity of 98.82%. Furthermore, the SDT (Sequence Demarcation Tool) triangular matrix revealed that the pairwise amino acid identities among intra-subfamily sequences within *Heptrevirinae* ranged from 37.30% to 100.00%, while those within *Ensavirinae* ranged from 52.10% to 100.00% (**Figure 2D**).

### 3.3 CRESS-DNA viruses

Among the DNA viruses detected in yak feces, CRESS-DNA viruses represented the most abundant and diverse group, including families such as *Circoviridae*, *Genomoviridae*, and *Smacoviridae*.

#### 3.3.1 Circoviridae

**Figure 3:**
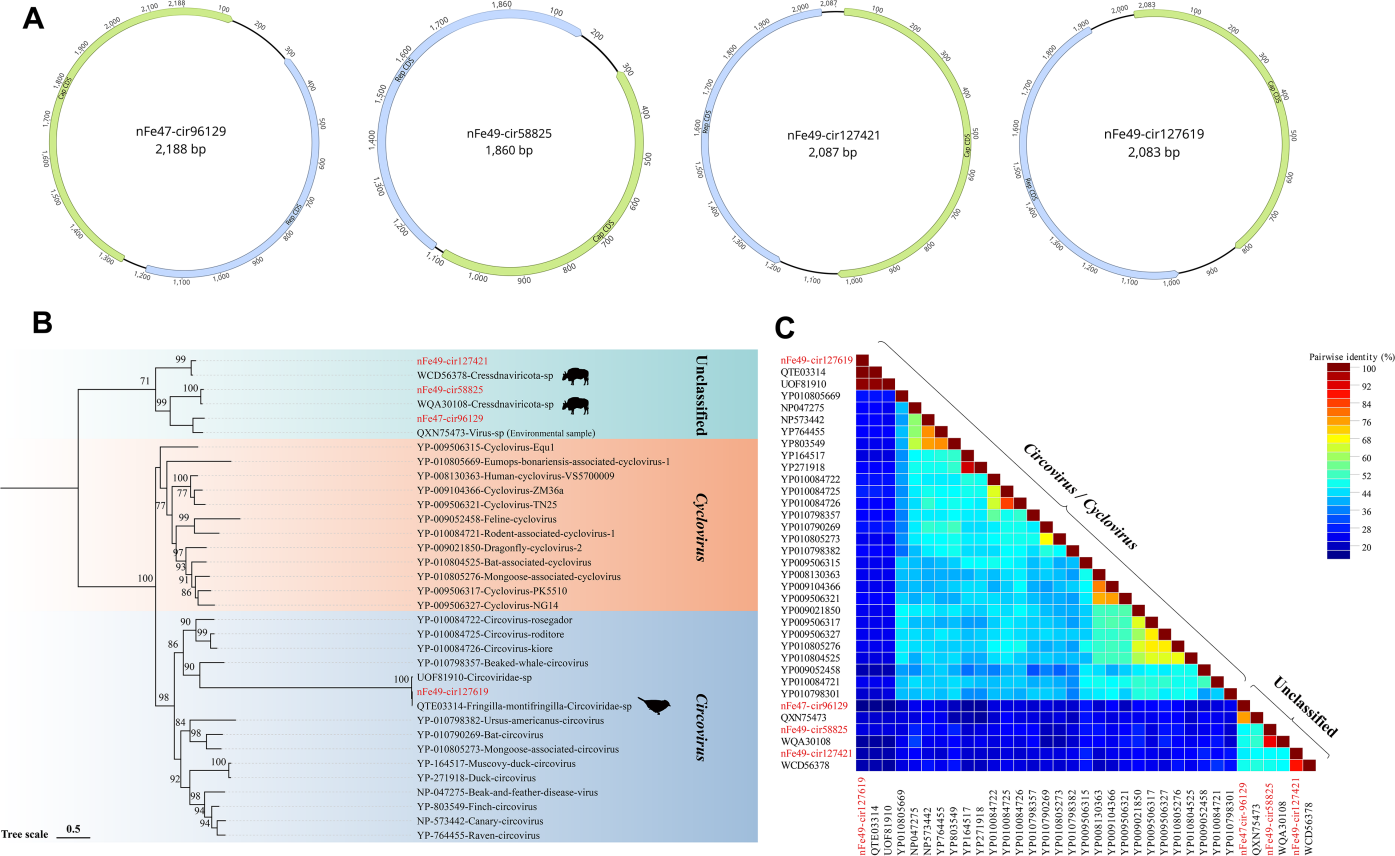
Phylogenetic Analysis of the *Circoviridae* Family. **(A)** Genomic structures of the four circoviruses identified in yak feces, with green ORFs representing those encoding the Cap protein and blue ORFs representing those encoding the Rep protein. **(B)** Phylogenetic tree constructed based on the Rep protein. Color blocks represent different viral genera, sequences identified in this study are marked in red, and Bootstrap values > 70 are displayed on the branches. The scale bar indicates the number of amino acid substitutions per site. **(C)** Triangular matrix heatmap showing the pairwise amino acid identity between sequences.

Circoviruses are single-stranded DNA (ssDNA) viruses and represent the smallest known animal viruses. The *Circoviridae* family is classified into two genera, *Circovirus* and *Cyclovirus*, which mainly encode replication-associated proteins (Rep) and capsid proteins (Cap), with a genome length of approximately 1.7–2.1 kb (Breitbart et al., 2017). In this study, four complete circovirus genomes were identified from the 9 libraries. The Rep and Cap proteins of these genomes are oriented in opposite directions, and the full-length genomes of the four strains are 2188 bp, 1860 bp, 2087 bp, and 2083 bp, respectively (**Figure 3A**). A phylogenetic tree was constructed based on the Rep protein (**Figure 3B**). The results showed that strain nFe49-cir127619 clustered with reference sequences of the *Circovirus* genus in the GenBank database, and it shared the closest genetic relationship with a strain derived from *Fringila montifringilla* (GenBank Accession No. QTE03314) in terms of the Rep protein gene, with an amino acid identity of 100.00%. The remaining three strains formed an independent clade: strain nFe49-cir127421 clustered with a cattle-derived strain (GenBank Accession No. WCD56378) (amino acid identity: 88.42%); strain nFe49-cir58825 clustered with a *Bos mutus*-derived strain (GenBank Accession No. WQA30108) (amino acid identity: 89.23%); and strain nFe47-cir96129 clustered with a strain derived from atmospheric particulate matter (GenBank Accession No. QXN75473) (amino acid identity: 76.53%). Furthermore, the SDT triangular matrix revealed that the pairwise amino acid identities among sequences within the *Circovirus*/*Cyclovirus* genera ranged from 19.00% to 100%, while the pairwise amino acid identities among viral sequences that did not cluster with known genera ranged from 46.00% to 90.40% (**Figure 3C**). Moreover, canonical stem-loop structures were identified in the 5’ intergenic regions of all four genomes (**Supplementary Figure 3**). Specifically, three genomes encode the conserved nonanucleotide motif TAGTATTAC, whereas strain nFe49-cir127619 harbors a single-nucleotide variant, TACTATTAC. The presence of these replication origin elements confirms the integrity and circularity of the assembled genomes.

#### 3.3.2 Genomoviridae

**Figure 4:**
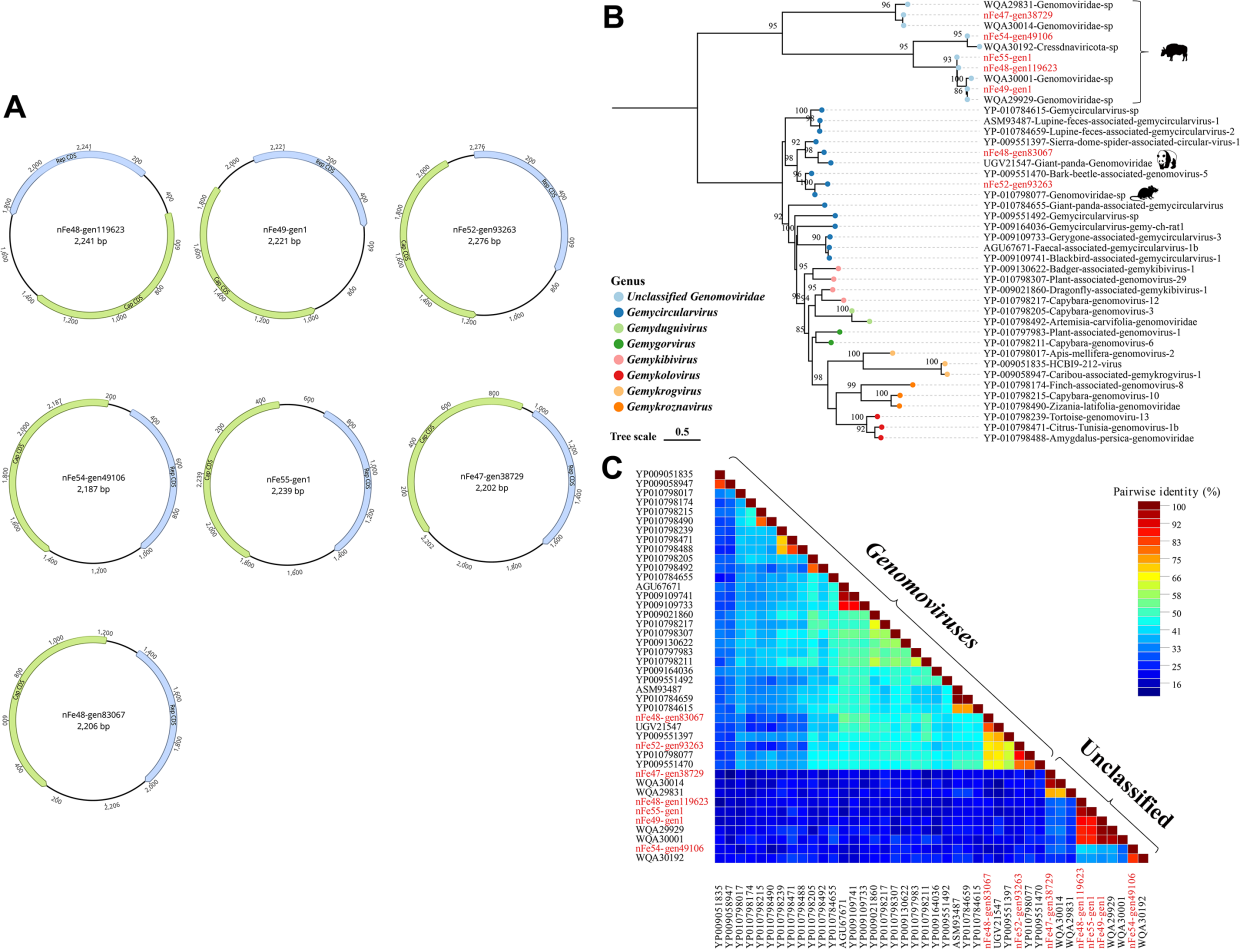
Phylogenetic Analysis of the *Genomoviridae* Family. **(A)** Genomic structures of the seven genomoviruses identified in yak feces, with green ORFs representing those encoding the Cap protein and blue ORFs representing those encoding the Rep protein. **(B)** Phylogenetic tree constructed based on the Rep protein. Sequences identified in this study are marked in red, and the legend is located at the bottom left. Circular color blocks represent different viral genera; Bootstrap values > 70 are displayed on the branches. The scale bar indicates the number of amino acid substitutions per site. **(C)** Triangular matrix heatmap showing the pairwise amino acid identity between sequences.

The *Genomoviridae* family primarily encodes capsid proteins (Cap) and replication-associated proteins (Rep), with most of its viral genomes having been sequenced from diverse environmental, animal, or plant specimens (Varsani and Krupovic, 2021). In this study, a total of 7 complete genomes were assembled from the 9 libraries. The Rep and Cap proteins of these genomes are oriented in opposite directions, with genome lengths ranging from 2187 to 2276 bp (**Figure 4A**). A phylogenetic tree was constructed based on the Rep protein (**Figure 4B**). The results showed that strains nFe48-gen83067 and nFe52-gen93263 clustered with viral protein sequences belonging to the *Gemycircularvirus* genus. Specifically, these two strains grouped with a panda-derived viral protein sequence (GenBank Accession No. UGV21547) and a mouse-derived viral protein sequence (GenBank Accession No. YP_010798077), respectively, with amino acid identities of 81.34% and 97.46%. In addition, the remaining 5 strains—nFe47-gen38729, nFe54-gen49106, nFe55-gen1, nFe48-gen119623, and nFe49-gen1—all clustered with yak-derived viral protein sequences, forming an independent clade, with amino acid identities ranging from 87.16% to 98.90%. The SDT triangular matrix revealed that the pairwise amino acid identities among viral protein sequences belonging to known genomoviruses ranged from 25.30% to 99.70%, while the pairwise amino acid identities among viral protein sequences that did not cluster with known genera ranged from 26.70% to 98.90% (**Figure 4C**).

#### 3.3.3 Smacoviridae

**Figure 5:**
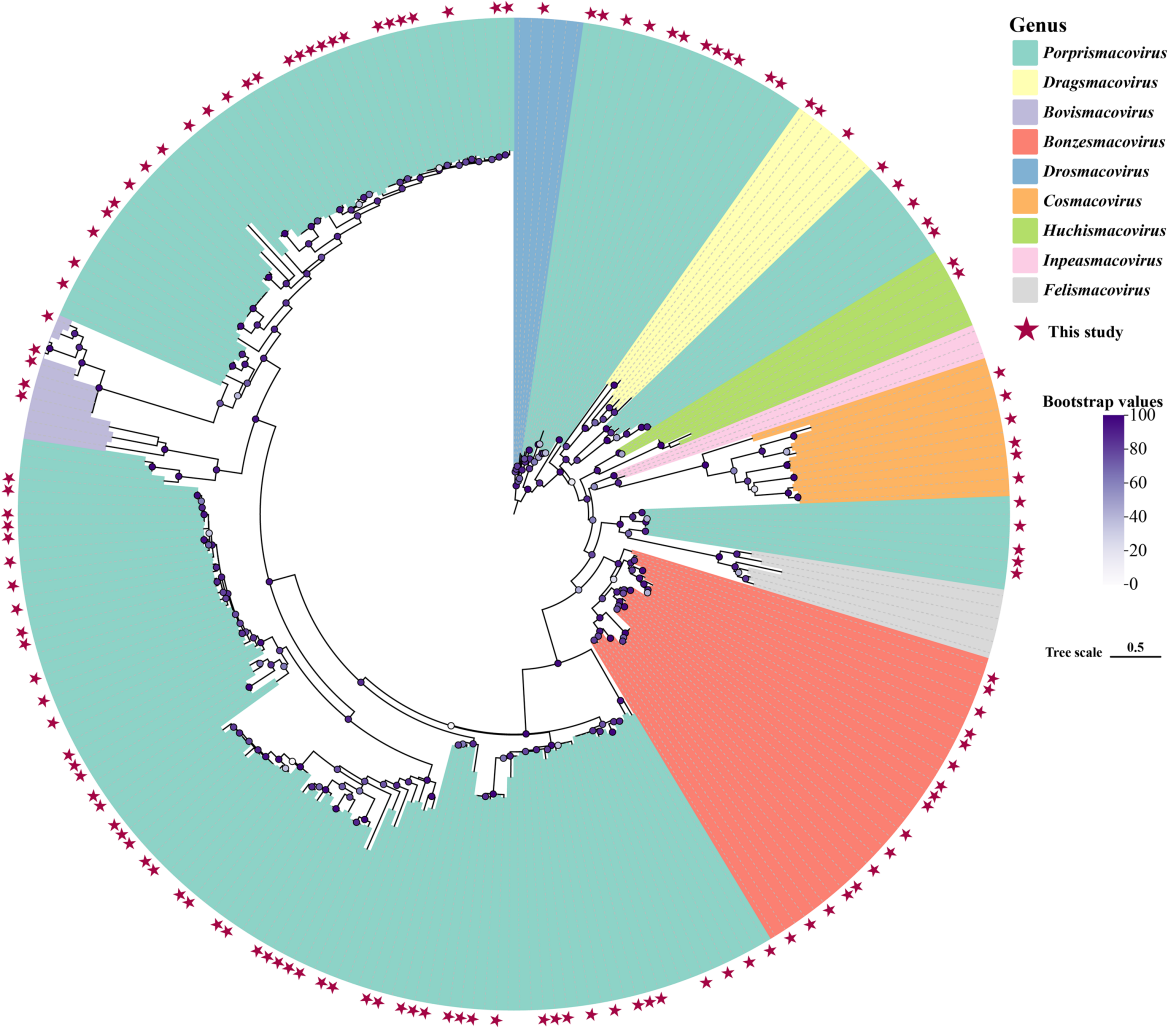
Phylogenetic Analysis of the *Smacoviridae* Family. Color blocks represent different viral genera, red pentagrams indicate sequences identified in this study, and the legend for Bootstrap values is located on the right side. The scale bar indicates the number of amino acid substitutions per site.

Viruses in the *Smacoviridae* family have a circular genome with a length of approximately 2.3–3.0 kb and can infect nearly all eukaryotic organisms (Varsani and Krupovic, 2024). In this study, a total of 185 complete genome sequences were identified from the 9 libraries. A phylogenetic tree was constructed based on the Rep protein (**Figure 5**), and the results showed that, compared with known viruses, the vast majority of these viral genome sequences shared sequence similarity with yak-derived smacoviruses, clustering with different genera, with amino acid identities ranging from 57.09% to 100.00%.

Recent studies have defined the criteria for new species and genera within *Smacoviridae*: new species are those with genome-wide identity < 77%, and new genera are those with Rep protein identity < 40% (Varsani and Krupovic, 2024). Therefore, the 185 viruses identified in this study do not belong to new species or genera. Additionally, the SDT triangular matrix constructed from these 185 *Smacoviridae* sequences revealed that these sequences are distributed across different genera and form multiple clusters (**Supplementary Figure 4**).

## 4 Discussion

With the continuous advancement of metagenomics and high-throughput sequencing technologies, the composition and evolutionary characteristics of the mammalian fecal virome are being gradually uncovered. Not only are gut viruses an important component of the host’s microecosystem, but they also play potential roles in host health, environmental adaptation, and the balance of microbial communities (Shkoporov et al., 2022; Yu et al., 2025). Through the systematic analysis of fecal metagenomes from yaks on the Tibetan Plateau, this study comprehensively depicts the compositional characteristics and phylogenetic relationships of their fecal viral communities, providing new insights into the viral diversity of plateau animals and their ecological and evolutionary patterns. Importantly, the observed viral diversity has significant ecological and evolutionary implications, as it reflects the adaptation of viruses to the extreme environmental conditions of the Tibetan Plateau, including low temperature, hypoxia, and high UV radiation. Such adaptations may influence viral stability, persistence, and interactions with host microbiota, thereby shaping the overall gut ecosystem and potentially contributing to host survival in this challenging environment (Zhu Y. et al., 2024).

Rarefaction and accumulation curves tended to plateau, indicating that under the current sample size and sequencing depth, the most abundant viral taxa within the pools have been sufficiently captured, and the obtained results have good representativeness at the macro level. However, metagenomic sequencing still has limited coverage of low-abundance viruses, especially RNA viruses, which may be undetected due to their high degradability and database bias (Callanan et al., 2021; Haagmans et al., 2025). Thus, the results of this study mainly reflect the overall structure of relatively high-abundance viral communities in the yak feces.

In terms of community composition, the yak fecal virome composition in our libraries is characterized by a high abundance of single-stranded DNA viruses belonging to the CRESS-DNA group, mainly including *Smacoviridae*, *Circoviridae*, and *Genomoviridae*. These viruses have small genomes, high stability, and low sensitivity to environmental degradation, making them easily retained during sample processing and enrichment (Sommers et al., 2019; Wang et al., 2022). CRESS-DNA viruses exhibit strong tolerance: they can maintain infectivity in low-temperature and arid environments, and even persist in permafrost for long periods, thus becoming an important component of the viral ecological cycle on the plateau (Liu et al., 2020; Wu et al., 2022).

In contrast, positive-sense single-stranded RNA (+ssRNA) viruses, mainly belonging to *Picornaviridae*, were detected at a significantly lower proportion. The two assembled Picornavirus genomes in this study showed high homology with ruminant-associated viruses, and phylogenetic analysis based on RdRp indicated that they clustered with yak- or deer-related viruses—suggesting these viruses may have long adapted to the enteric ecological environment of ruminant hosts (Pan et al., 2023). Notably, some viral sequences formed closely related clades with strains derived from other ruminants, implying potential networks of viral sharing or cross-species transmission among different animal populations in the plateau region (Carrascosa-Sàez et al., 2024). This transmission pattern may be facilitated through pathways such as forage, water sources, or fecal contamination, reflecting the complexity of viral spread in the open grassland ecosystem. Of course, the detection rate of RNA viruses in this study was relatively low (0.71%). Due to their generally high mutation rates and short environmental persistence times, the detection of RNA viruses may be influenced by multiple factors, including sample degradation, library construction strategies, and reverse transcription efficiency (Duffy, 2018; Gu et al., 2019). However, a low abundance of RNA viruses does not imply weak ecological significance (Starr et al., 2019). Previous studies have shown that certain low-abundance RNA viruses may play key roles in intestinal ecological balance and host immune regulation through transient infection or co-infection mechanisms (Wille et al., 2018). Therefore, the true diversity of RNA viruses may be underestimated, and future studies should adopt RNA enrichment and metatranscriptomic sequencing strategies to more comprehensively capture their activity signals (Ogunbayo et al., 2023).

Among CRESS-DNA viruses, both *Circoviridae* and *Genomoviridae* exhibited high sequence diversity. Among the four *Circoviridae* viral genomes identified in this study, some showed 100% amino acid identity with the Rep proteins of avian-derived viruses, suggesting the possibility of cross-species transmission (Kaszab et al., 2020). Birds have a wide range of activities and frequent migrations in the plateau ecosystem, and may participate in the ecological spread of viruses through feces or arthropod vectors (Cui et al., 2011). Furthermore, some sequences clustered with viruses associated with atmospheric particulate matter, indicating potential genetic relevance to atmospheric particle samples. Collectively, these findings suggest that in the open and dynamic environment of the Tibetan Plateau, viruses not only undergo intra-host circulation but may also achieve cross-environment transmission through air, soil, and permafrost (Vidovszky et al., 2023; Zhu P. et al., 2024).

It is important to acknowledge that detecting viral sequences via metagenomics does not equate to confirming active infection or host colonization. A primary limitation of our study is the absence of experimental validation for viral replication, such as the detection of replicative intermediates or viral-host co-localization assays (e.g., FISH) (Vyboh et al., 2012). Consequently, we cannot rule out the possibility that some identified taxa, particularly those in low abundance, are transient ‘passengers’ introduced through forage or environmental exposure rather than established residents of the yak enteric ecosystem. As such, the viral diversity described here is best characterized as a ‘fecal-associated virome, ‘ and future studies incorporating functional assays are requisite to definitively establish host-virus associations.

For viral sequences from the *Genomoviridae* family, most strains were distributed within the existing viral family and genus framework, but several independent evolutionary clades still existed. These clades may represent undescribed viral lineages, reflecting the trend of viral diversification in the Tibetan Plateau ecosystem. Such phenomena are particularly common in extreme environments—region-specific viral communities have been detected in Antarctic dry valleys (Zhang et al., 2025), Arctic permafrost (Otieno et al., 2021b), and plateau lakes (Zhu et al., 2022). In addition, environmental isolation and low-temperature conditions may limit genetic exchange between viruses and the external environment, thereby facilitating the formation of locally specific lineages (Adriaenssens et al., 2017; Manuel and Snyder, 2024). Meanwhile, the long-term low temperature, hypoxia, and high radiation conditions on the Tibetan Plateau provide continuous natural selection pressure for viral evolution, endowing viruses with stable capsid structures, compact genomes, and high GC content with greater survival advantages (Gil et al., 2021; Ettinger et al., 2023).

A particularly prominent large-scale divergence was observed in the *Smacoviridae* family, whose phylogenetic tree exhibited characteristics of multiple branches and extensive differentiation—indicating that they may have undergone significant lineage expansion during evolution. Moreover, these viruses possess non-enveloped circular genomes, enabling them to resist desiccation and low temperatures and potentially maintain long-term activity in plateau permafrost or feces (Krupovic and Varsani, 2021).

Current database alignments did not reveal known pathogenic motifs or functional markers relevant to vertebrate hosts in the viral genomes identified here. However, the absence of annotated virulence factors does not preclude pathogenic potential. Without supporting functional predictions, transcriptomic profiles, or host health metadata, ecological interpretations of these viruses remain speculative. We thus posit that they likely represent putative commensal or environmentally adapted members of the yak fecal virome, a conclusion that warrants confirmation through future functional characterization.

It is worth emphasizing that this study has continuity in research direction with our previously published yak virome study in *Communications Biology* (DOI: 10.1038/s42003-024-06798-y), but there are significant differences in data sources, research focuses, and analytical levels. The datasets of the two studies are mutually independent, corresponding to different BioProjects (PRJCA018020/PRJNA994540 and PRJCA039468), and the sampling regions and times do not overlap. This study did not reuse any previous data or assembly results; instead, an independent metagenomic database was constructed based on new samples. In contrast to the previous study, which focused on the macro-distribution pattern of yak viral communities across different regions, this study pays more attention to the genomic characteristics and phylogenetic relationships of specific viral taxa in individual samples—particularly at the level of CRESS-DNA viruses and *Picornaviridae*. Several nearly complete genomic sequences were identified, significantly expanding the known genomic range of yak-associated viruses. The newly added *Smacoviridae* and *Genomoviridae* lineages not only supplement the relatively scarce circular DNA virus information in previous reports but also provide a new reference framework for further comparative studies on ruminant enteric viral diversity. Additionally, the *Picornaviridae* and CRESS-DNA viruses revealed in this study exhibit higher phylogenetic diversity. Their positions in the phylogenetic tree show close relationships with ruminant viruses, along with signs of independent evolution in several clades. These results suggest that under similar ecological contexts, viral communities may have undergone long-term host adaptation and environmental filtering.

Together, these observations highlight the important ecological and evolutionary consequences of viral diversity on the Tibetan Plateau, reflecting both host adaptation and environmental selective pressures that shape the fecal virome structure. The findings of this study complement the earlier yak virome results: the former focuses on the macro-description of ecological distribution and community patterns, while this study further enriches the genomic information and evolutionary clues of specific viral taxa. Together, they construct a more comprehensive ecological map of the yak fecal virome.

Although this study has revealed the composition and potential ecological connections of the yak fecal viral community, it still has certain limitations. First, the pooling strategy (43 samples into 9 libraries) was employed to maximize viral discovery breadth. However, we acknowledge that this approach masks individual-level heterogeneity. Consequently, high viral abundance in a library could result from either high prevalence across the population or high viral shedding in a few individuals. Thus, our data reflect the ‘community aggregate’ rather than individual prevalence. Furthermore, this design precludes the analysis of viral-host associations based on individual metadata and limits our ability to construct precise co-occurrence networks between specific viruses and bacteria. Our study utilizes a cross-sectional design based on sampling at a single time point (October). We acknowledge that this approach inherently limits our ability to capture the temporal dynamics or seasonal stability of the yak fecal virome. While this dataset establishes a critical baseline for viral diversity in this specific season, it does not reflect longitudinal fluctuations driven by seasonal dietary changes or climate variations. Future studies should consider employing individual-level samples for more detailed virome analyses, so as to more comprehensively understand the impacts of host specificity and individual differences on the viral community. In addition, due to the inherent biases of the metagenomic approach employed in this study for RNA virus detection, low-abundance RNA viruses may not be fully captured (Manso et al., 2017; Wu et al., 2024). The high mutation rate and short environmental persistence of RNA viruses result in their potentially weak presence in samples, especially when samples undergo degradation or exhibit low reverse transcription efficiency (Donaghy and Jupp, 1995). Therefore, future studies can overcome this limitation through RNA enrichment techniques and transcriptomic methods, so as to more comprehensively reveal the diversity and functions of low-abundance RNA viruses. Additionally, the dataset of this study comprises pooled samples, lacking individual-level host information, which restricts in-depth analysis of host-virus associations. Analyses at the individual host level can reveal more nuanced ecological relationships between hosts and viruses (Kinsella et al., 2020). In future research, integrating host metadata to conduct higher-level analyses of host-virus interactions will help explore the potential impacts of host specificity on viral communities. Meanwhile, future studies can further investigate the mechanisms by which environmental gradients influence the enteric viral communities of plateau animals through cross-species comparisons.

## 5 Conclusion

In summary, based on an independent metagenomic dataset, this study expanded the genomic resources of the fecal virome of yaks on the Tibetan Plateau and delineated the diversity and potential origins of several key viral taxa through phylogenetic analysis. The results not only supplement the previous yak virome study at the data level but also provide new insights at the phylogenetic and taxonomic levels. Given the limitations of samples and methods, we remain cautious about inferences regarding ecological and functional aspects. Future studies should be conducted across a broader spatiotemporal and host range, integrating strategies such as individual-level sequencing and metatranscriptomics to further clarify the true roles and evolutionary significance of these viruses in yaks and their ecosystems.

## Data Availability

The datasets presented in this study can be found in online repositories. The names of the repository/repositories and accession number(s) can be found below: Raw sequencing data from this study are available in the Genome Sequence Archive (GSA) (Chen et al., 2021) in National Genomics Data Center (NGDC), China National Center for Bioinformation / Beijing Institute of Genomics, Chinese Academy of Sciences, under BioProject accession number PRJCA039468 (https://ngdc.cncb.ac.cn/gsa). Experimental datasets are deposited in GenBase (Bu et al., 2024) (accession numbers C_AA107965.1–C_AA108162.1) at https://ngdc.cncb.ac.cn/genbase.

## Supplementary material

Supplementary Figure 1 Yak fecal sample collection site.

Supplementary Figure 2 Beta diversity analysis of viral communities. Principal Coordinate Analysis (PCoA) plot based on Bray-Curtis dissimilarity, visualizing the compositional differences among the nine yak fecal metagenomic libraries. The percentage of variation explained by the first two principal coordinates (PCo1 and PCo2) is indicated on the axes.

Supplementary Figure 3 Predicted stem-loop structures of the four identified circoviruses. The secondary structures are displayed based on the reverse complement strand. Consequently, the apical loops exhibit the sequence GTAATACTA (corresponding to the canonical nnanucleotide motif TAGTATTAC) or the variant GTAATAGTA (corresponding to TACTATTAC in strain nFe49-cir127619).

Supplementary Figure 4 Triangular matrix heatmap of amino acid identity from pairwise alignment between *smacoviridae* family sequences.

Supplementary Table 1 Overview of metagenomic samples, sequencing libraries, and metadata.

Supplementary Table 2 Alpha diversity indices of viral communities in the nine yak fecal libraries.

Supplementary Table 3 Overview of viral strains analyzed in this study.
