# Supplementary figures and images for: Viral metagenomic analysis of fecal samples from Bos grunniens on the Qinghai-Tibet Plateau reveals novel picornaviruses and diverse CRESS-DNA viruses
Source: Front Cell Infect Microbiol. 2026 Jan 7;15:1719300. doi: 10.3389/fcimb.2025.1719300 (PMC12819777; doi:10.3389/fcimb.2025.1719300)

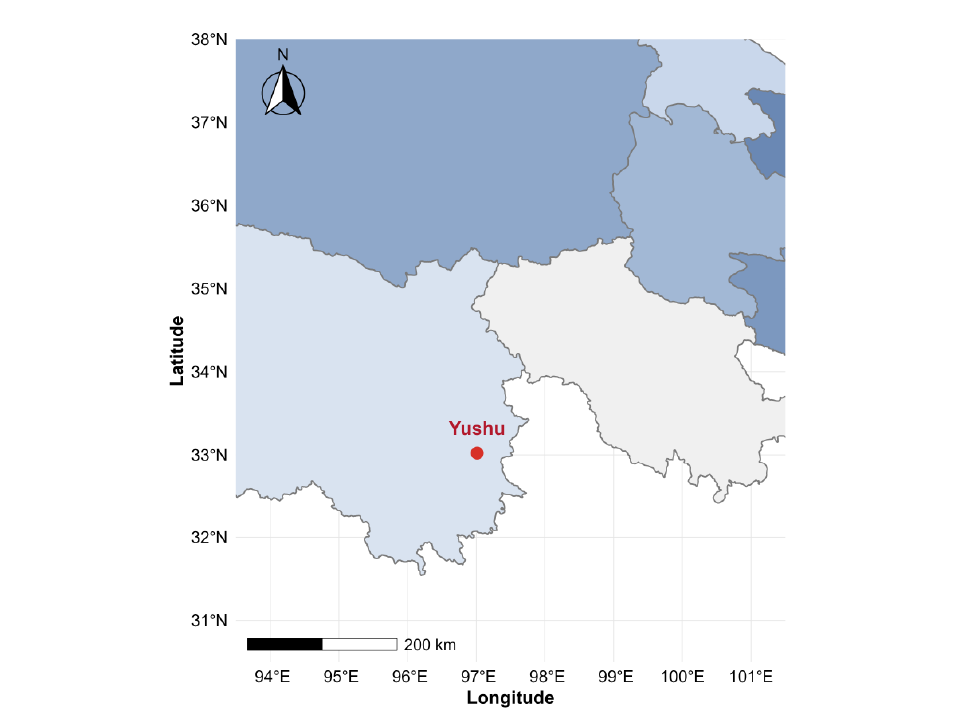

Supplement: Supplementary Figure 1 — Yak fecal sample collection site. [file Image1.tif]

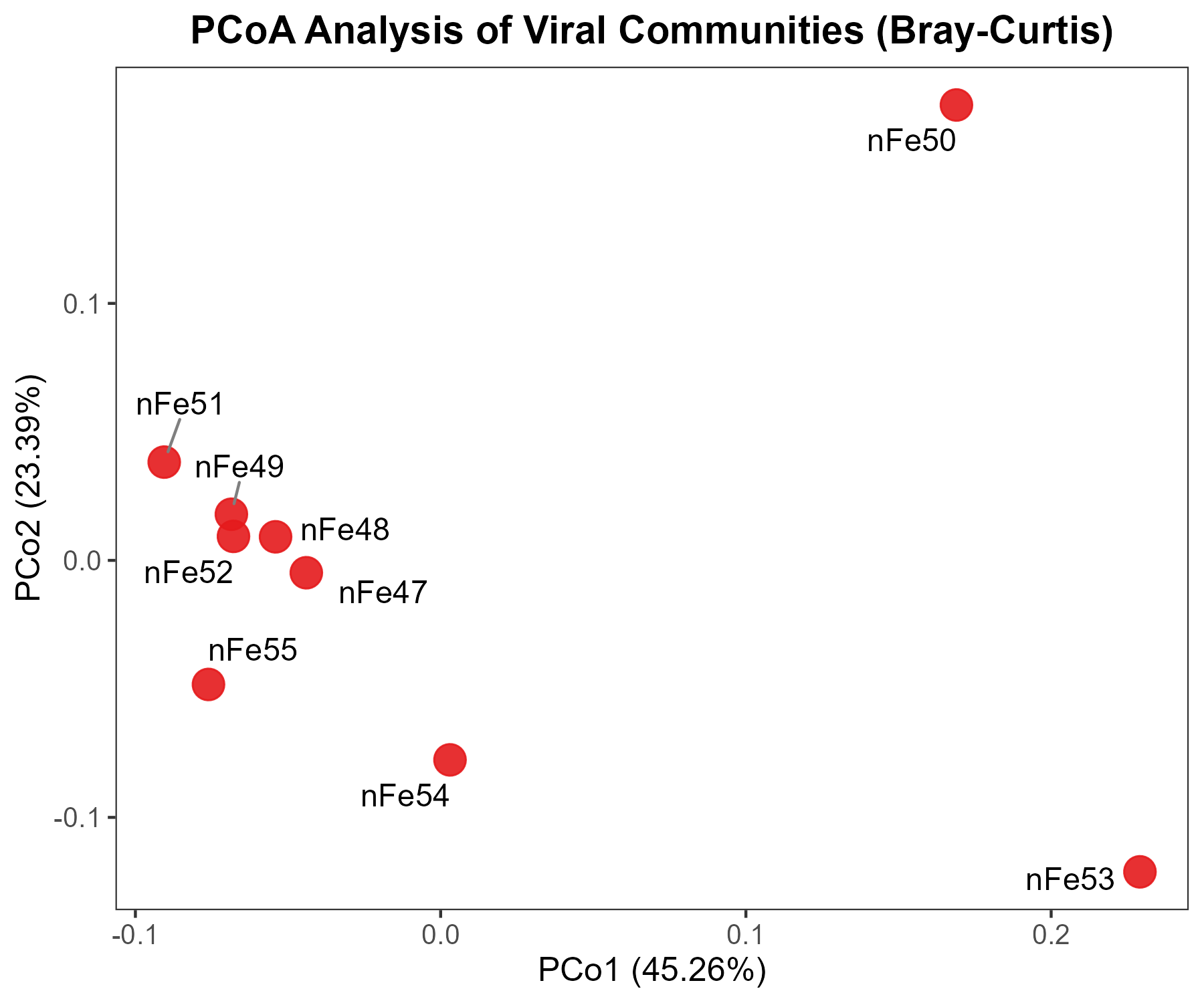

Supplement: Supplementary Figure 2 — Beta diversity analysis of viral communities. Principal Coordinate Analysis (PCoA) plot based on Bray-Curtis dissimilarity, visualizing the compositional differences among the nine yak fecal metagenomic libraries. The percentage of variation explained by the first two principal coordinates (PCo1 and PCo2) is indicated on the axes. [file Image2.png]

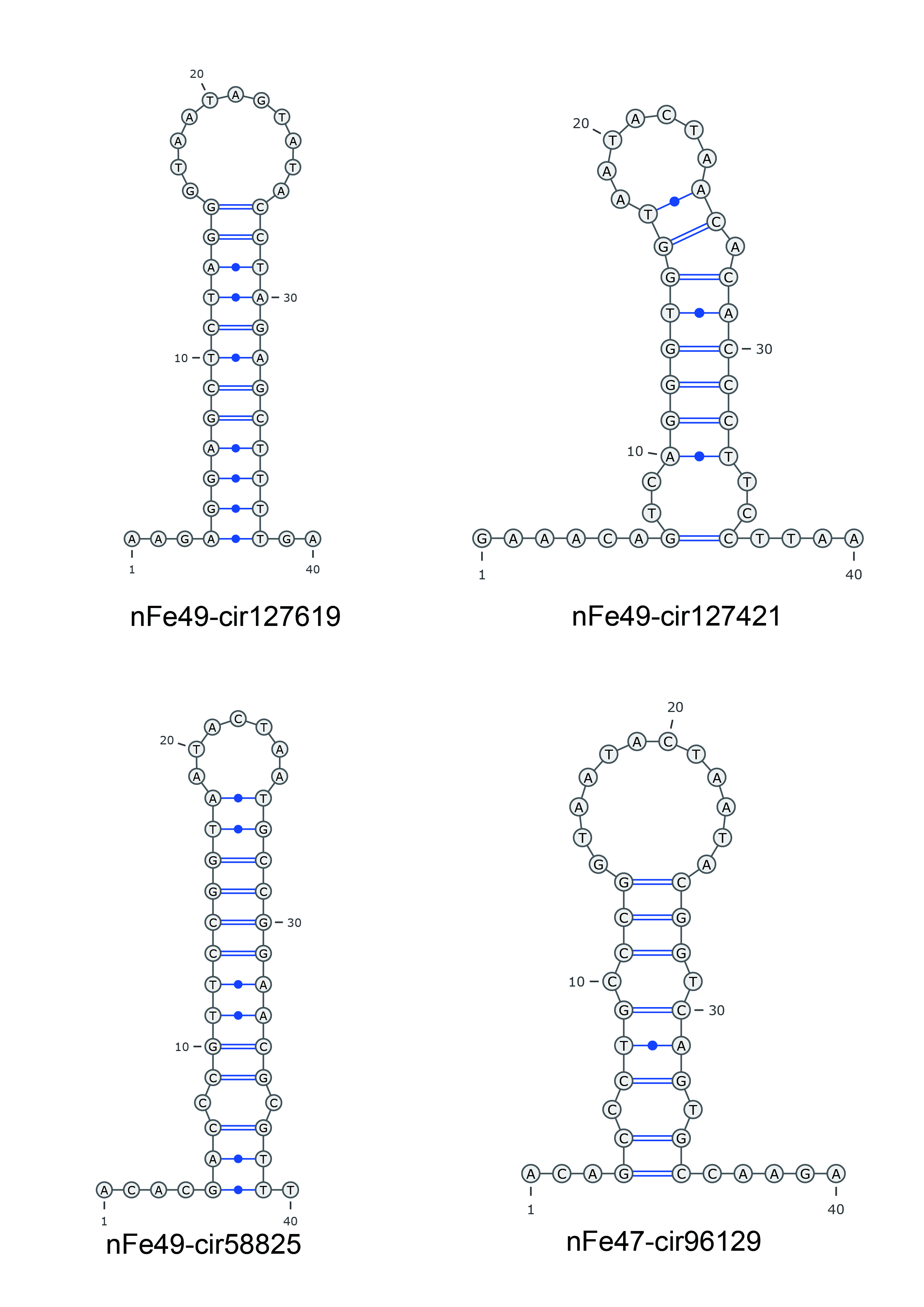

Supplement: Supplementary Figure 3 — Predicted stem-loop structures of the four identified circoviruses. The secondary structures are displayed based on the reverse complement strand. Consequently, the apical loops exhibit the sequence GTAATACTA (corresponding to the canonical nnanucleotide motif TAGTATTAC) or the variant GTAATAGTA (corresponding to TACTATTAC in strain nFe49-cir127619). [file Image3.tif]

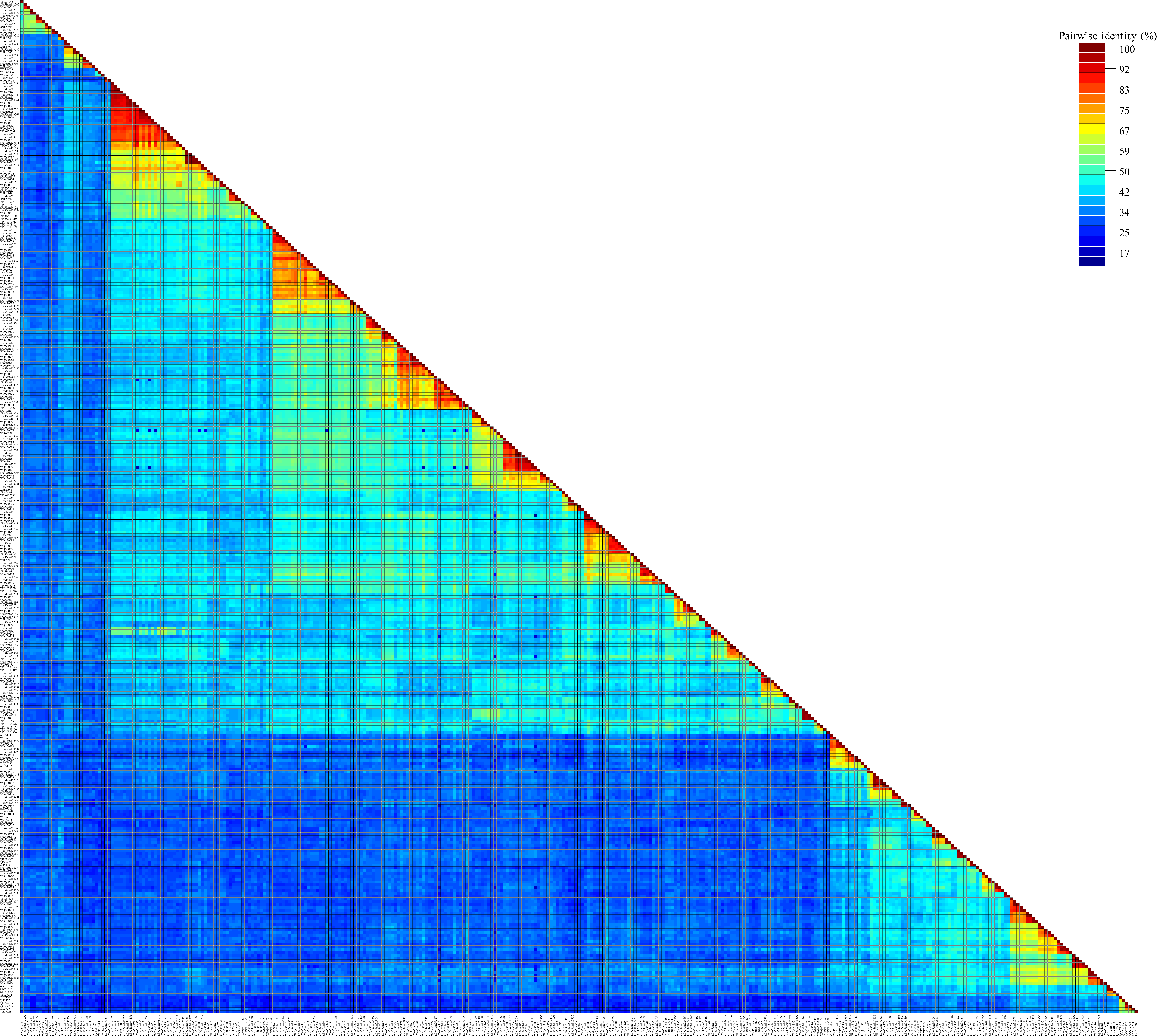

Supplement: Supplementary Figure 4 — Triangular matrix heatmap of amino acid identity from pairwise alignment between smacoviridae family sequences. [file Image4.tif]
