# Supplementary material for: Viral metagenomic analysis of fecal samples from Bos grunniens on the Qinghai-Tibet Plateau reveals novel picornaviruses and diverse CRESS-DNA viruses
Source: Front Cell Infect Microbiol. 2026 Jan 7;15:1719300. doi: 10.3389/fcimb.2025.1719300 (PMC12819777; doi:10.3389/fcimb.2025.1719300)
Supplement: Supplementary Table 2 — Alpha diversity indices of viral communities in the nine yak fecal libraries. [file Table2.docx]

**Alpha diversity indices of viral communities in the nine yak fecal libraries.**

| Sample ID | Observed Species | Shannon Index | Simpson Index |
| --- | --- | --- | --- |
| nFe47 | 45 | 2.83 | 0.88 |
| nFe48 | 41 | 2.78 | 0.88 |
| nFe49 | 47 | 2.81 | 0.87 |
| nFe50 | 47 | 2.91 | 0.91 |
| nFe51 | 43 | 2.76 | 0.87 |
| nFe52 | 40 | 2.67 | 0.87 |
| nFe53 | 49 | 3.08 | 0.92 |
| nFe54 | 45 | 2.84 | 0.89 |
| nFe55 | 49 | 2.84 | 0.88 |
